# Compensation for radiotherapy treatment interruptions due to a cyberattack: An isoeffective DVH‐based dose compensation decision tool

**DOI:** 10.1002/acm2.13716

**Published:** 2022-07-20

**Authors:** Katie O'Shea, Linda Coleman, Louise Fahy, Christoph Kleefeld, Mark J. Foley, Margaret Moore

**Affiliations:** ^1^ School of Physics National University of Ireland Galway Galway Ireland; ^2^ Department of Medical Physics and Clinical Engineering University Hospital Galway Galway Ireland

**Keywords:** cyberattack, dose compensation, dose–volume histograms, radiobiological dose, treatment interruptions

## Abstract

Unscheduled interruptions to radiotherapy treatments lead to decreased tumor control probability (TCP). Rapid cell repopulation in the tumor increases due to the absence of radiation dose, resulting in the loss of TCP. Compensation for this loss is required to prevent or reduce an extension of the patient's overall treatment time and regain the original TCP. The cyberattack on the Irish public health service in May 2021 prevented radiotherapy treatment delivery resulting in treatment interruptions of up to 12 days. Current standards for treatment gap calculations are performed using the Royal College of Radiologists (RCR) methodology, using a point‐dose for planning target volume (PTV) and the organs at risk (OAR).

An in‐house tool, named EQD_2_VH, was created in Python to perform treatment gap calculations using the dose–volume histogram (DVH) information in DICOM data extracted from commercial treatment planning system plans. The physical dose in each dose bin was converted into equivalent dose in 2‐Gy fractions (EQD_2_), accounting for tumor cell repopulation. This EQD_2_‐based DVH provides a 2D representation of the impact of treatment gap compensation strategies on both PTV and OAR dose distributions compared to the intended prescribed treatment plan. This additional information can aid clinicians’ choice of compensation options. EQD_2_VH was evaluated using five high‐priority patients experiencing a treatment interruption when the cyberattack occurred. Compensation plans were created using the RCR methodology to evaluate EQD_2_VH as a decision‐making tool.

The EQD_2_VH method demonstrated that the comparison of compensated treatment plans alongside the original intended treatment plans using isoeffective DVH analysis can be achieved. It enabled a visual and quantitative comparison between treatment plan options and provided an individual analysis of each structure in a patient's plan. It demonstrated potential to be a useful decision‐making tool for finding a balance between optimizing dose to PTV while protecting OARs.

## 1 INTRODUCTION

Treatment gaps in radiotherapy are unscheduled interruptions in a patient's treatment schedule that can lead to a decrease in the tumor control probability. They are a common occurrence in radiotherapy treatments, with 63% of head‐and‐neck cancer patients experiencing at least one treatment gap.^1^, ^2^ Interruptions to treatments can be caused by the servicing or breakdown of machinery, patient illnesses preventing treatments, and public holidays.^2^ Advancements in radiotherapy have resulted in an increased reliance on technology for the delivery of treatments. The complete loss of access to electronic patient data and electronic control of medical devices, such as linear accelerator (linac) record and verify systems, due to a cyberattack is a new source of interruption to the delivery of radiotherapy treatments. The increasing prevalence of healthcare cyberattacks makes radiotherapy a vulnerable target.^3^

In June 2021, the United States Department of Health and Human Services reported 82 cyberattacks on healthcare sectors worldwide in the first 5 months of 2021 alone.^4^ Two examples of healthcare cyberattacks in 2021 are the ransomware cyberattacks on the Waikato District Health Board in New Zealand, which resulted in the shutdown of radiotherapy treatments for 20 days, and the Department of Health and Health Service Executive (HSE) in Ireland that caused a 12‐day interruption to radiotherapy treatments. Treatment gaps of these durations can cause significant setbacks in a patient's treatment.^1^

The onset of rapid cell repopulation in tumor tissue during treatment gaps can result in a decrease in the radiobiological dose to the planning target volume (PTV). The standard biologically effective dose (BED) to the PTV and organs at risk (OARs) is calculated as follows^2^:

(1) $$\;BED_{PTV}=\;N\;\times \;d\;\times \;\left(1+\;\frac{d}{\frac{\alpha }{\beta }}\right)-K\;\times \;\left(T-\;T_{delay}\right)\;$$

(2) $$\;BED_{OAR}=\;N\;\times \;d\;\times \;\left(1+\;\frac{d}{\frac{\alpha }{\beta }}\right)$$

where *N* is the number of fractions, *d* is the physical dose per fraction (Gy), *T_delay_* is the time in calendar days between the beginning of treatment and when rapid cell repopulation begins in the tumor, *K* is the dose lost per day due to rapid cell repopulation (Gy day^−1^), and *T* is the overall treatment time in calendar days. The α/αββ is a tissue‐specific parameter, which represents the sensitivity of that tissue to dose fractionation.^5^ The values for *K* and *T_delay_* will depend on the tumor being treated, with fast growing tumors having a higher *K* and a shorter *T_delay_*.

An increase in *T* due to a treatment gap will result in a decrease in BED_PTV_; however, steps can be taken to minimize or prevent the extension onto the treatment time. The Royal College of Radiologists (RCR)^1^ published guidelines on managing treatment gaps, detailing the compensation methods that can be used to prevent an increase in *T*. These methods include increasing *N* while maintaining *T* by using twice‐daily fractionation or weekend treatments until the number of missed fractions have been compensated, and/or increasing *d* until the dose lost due to rapid cell repopulation during the gap has been compensated.^1^ Although these methods of compensation are successful in regaining the dose lost to the PTV, they do so at the expense of the OARs. Twice‐daily fractionation results in an increase in sublethal damage to normal tissue due to the incomplete repair of normal tissue between fractions, which increases the normal tissue complication probability (NTCP).^5^ Similarly, increasing *d* will increase the dose to the OARs and the NTCP. Although weekend treatments do not result in an increase in NTCP, they may not be suitable for all patients or offered by all treatment centers. The RCR recommends that the BED to the PTV and the OARs are calculated for each compensation option to assist decision‐making when choosing the compensation strategy most suitable for the patient.^1^

One limitation of the current use of RCR treatment gap calculations is the use of a point‐dose *d*, which is a 1D representation of the dose distribution within a volume. For the PTV, the prescription dose is used for the value of *d*, whereas for OARs, the RCR guidelines suggest using the dose actually received by the critical normal tissue if this is different from the prescribed dose.^1^ Typically, OARs have a nonuniform dose distribution, so the choice of a value of *d* in Equation (2) could be the near‐maximum dose (*D*_2%_) for the associated planning organ‐at‐risk volume as per ICRU Report 83 dose reporting guidelines.^6^ A worst case scenario approach for the most critical OAR prompts choosing the value of *d* at, or above, the maximum dose to this OAR, which leads to overestimation of the dose to this OAR. The calculations and compensation methods recommended by the RCR are also based on a standard treatment gap length of 4–5 days. The need for improved guidelines and calculations for prolonged treatment gaps occurring on a large scale was highlighted by Gay et al.^7^ after Hurricane Maria resulted in a 3‐week nationwide disruption to health services in Puerto Rico. The COVID‐19 pandemic and a cyberattack, such as the one on the HSE in Ireland on the 14 May 2021, resulted in large cohorts of patients unable to receive treatments for up to 12 days.^8^ Treatment gap calculations performed on patients during the cyberattack used the point‐dose method following RCR recommendations.

Current treatment gap dose compensation calculations can be improved upon by moving from a 1D, point‐dose representation to a 2D dose–volume representation of the effects that various compensation options produce for each individual structure. Dose–volume histograms (DVHs) display the physical dose generated by the treatment planning system (TPS) for each structure. Converting the physical dose output from the TPS‐based DVH into a radiobiological DVH for treatment gap calculations enables repopulation effects in the tumor tissue to be taken into account. This process provides a visual dose–volume analysis of compensation strategies and accounts for changes in dose fractionation, and inhomogeneous dose distributions in each structure. Further, converting the BED in Equations (1) and (2) into the equivalent dose in 2‐Gy fractionation (EQD_2_) for both the PTV and each OAR shows the total radiobiological dose needed to give the same biological effect assuming a conventional treatment schedule of 2 Gy per fraction.

(3) $$\;EQD_{2}=\;C\;\times \;BED$$

where $C=\frac{1}{\left(1+\frac{2}{\alpha /\beta }\right)}$, which is constant for a given tissue. Hence, conversion of the physical dose *D* to BED permits tumor tissue repopulation effects to be estimated (Equation 1), and the further conversion from BED to EQD_2_ (Equation 3) allows the addition of dose from subsequent compensation treatments that may have different prescribed dose per fraction regimes. Furthermore, clinicians are familiar with tissue tolerances expressed as EQD_2_ and this supports the decision‐making process.

This study reports the use of EQD_2_VH, an in‐house Python program created to convert the physical dose in DVHs into EQD_2_ while accounting for cell repopulation in the tumor. The use of EQD_2_VH was evaluated using revised plans consisting of accelerated fractionation and hypo‐fractionation to compensate for treatment gaps. Case studies were chosen from patients whose radiotherapy treatment was abruptly interrupted during the May 2021 cyberattack.

## 2 MATERIALS AND METHODS

### 2.1 Calculation method

The EQD_2_VH software program was created in Python using the Dicompyler library, an open‐source library that views and retrieves information from DICOM files.^9^ The RT Dose (RD) and RT Structure (RS) files from the original external beam radiotherapy (EBRT) treatment plans were required to retrieve the DVH information for each structure in the plan from the TPS. The parameters required by EQD_2_VH are presented in Table 1.

**TABLE 1:** Parameters required by EQD_2_VH

Parameter	Definition
α/β	The measure of the structure's sensitivity to fractionation
*T_delay_* (days)	Number of days after onset of treatment when rapid cell repopulation begins in the tumor
*K* (Gy day^−1^)	Rate of rapid cell repopulation in the tumor
*N_init_*	Number of fractions prescribed to the patient at the beginning of their initial treatment
*N_pre_*	Number of fractions completed before the interruption to their treatment
*N_post_*	Number of fractions completed after the interruption to their treatment
*T_init_* (days)	The initial overall treatment time
*T_rev_* (days)	The revised overall treatment time, including their treatment gap
*TD* (days)	The number of treatment days where twice‐daily fractionation was used

The physical TPS dose for the PTV and the OARs first needed to be converted into BED. This accounts for repopulation effects in the PTV and the effects of sublethal damage to unrepaired tissue during twice‐daily fractionation. The increase in damage to the tissue during twice‐daily fractionation is represented by *h*, which results in an apparent increase in the BED.^2^ This is a result of the increase in damage due to sublethal repair of tissue during closely spaced fractions. This is only valid for closely spaced fractions < 8 h apart. If the interfraction interval is > 8 h, then *h* = 0.

(4) $$\begin{array}{ccc} BED_{PTV} & = & \;N\;\times \;d\;\times \;\left(1+\;\frac{d\left(1+h\right)}{\alpha /\beta }\right)\\ & & -\;K\;\times \;\left(T-\;T_{delay}\right) \end{array}$$

(5) $$\;BED_{OAR}=\;N\;\times \;d\;\times \;\left(1+\;\frac{d\left(1+h\right)}{\alpha /\beta }\right)$$

The BED conversion was modified to account for dose variations in each structure using a variable‐dose method. The dose per fraction *d* was replaced by DDVHi/DDVHiNN, where $D_{DVH_{i}}$ is the physical dose in bin *i* of the DVH dataset containing *M* bins for the given structure:

(6) $$\begin{array}{ccc} BED_{PTV} & = & \sum_{i=1}^{M}D_{DVH_{i}}\;\;\times \;\left(1+\;\frac{\frac{D_{DVH_{i}}}{N}\left(1+h\right)}{\alpha /\beta }\right)\\ & & -\;K\;\times \;\left(T-\;T_{delay}\right) \end{array}$$

(7) $$\;BED_{OAR}=\;\sum_{i\;=\;1}^{M}D_{DVH_{i}}\times \;\left(1+\;\frac{\frac{D_{DVH_{i}}}{N}\left(1+h\right)}{\alpha /\beta }\right)\;$$

This modification iterates through each dose bin and uses the physical dose distribution present in the structure to calculate the variable dose per fraction. The BED for each structure was then converted into EQD_2_ using Equation (3) to normalize each treatment to conventional fractionation and to make it possible to sum plans with different fractionation schemes together.

Dicompyler accesses the DVH information from the RD file using the *dvhcalc.get_dvh* module.^10^ The module was modified to take in an additional parameter, RB_convert, which would be multiplied by *D_DVH_* to convert it into the radiobiological dose. As seen in Equations (6) and (7), the term that is multiplied by *D_DVH_* also contains *D_DVH_/N*. The DVH information for each structure was therefore obtained twice using *dvhcalc.get_dvh*. The first use assumed RB_convert = 1 to calculate the array of the physical dose per fraction *D_DVH_/N* in each dose bin. This *D_DVH_/N* array was then used in RB_convert to convert the corresponding *D_DVH_* into the EQD_2_ using Equation (3). The volume of each structure was normalized to the number of bins present and was then graphed with the radiobiological array of *D_DVH_*.

### 2.2 Software applications

The *dvhcalc.get_dvh* module also allowed the modification of the interpolation resolution *R*, which defines the resolution (in mm) of the dose grid to interpolate the dose data to, and the interpolation between segments *S*, which defines the number of segments to interpolate between CT slices. Several *R* values (*R* = 0.125, 0.375, 1 mm, and *R *= 0) and *S* values (*S* = 1, 2, 5 segments, and *S* = 0) were investigated to find which interpolation settings corresponded best with the Monaco HD (Elekta AB, Sweden) (version 5.5.1) TPS that was used for this project.

The radiobiological DVHs produced by EQD_2_VH were benchmarked against the DVHs obtained from Monaco for the same treatment plans for an independent verification of the software calculations. The DVHs from Monaco were manually converted into EQD_2_ using the same calculation equations used in EQD_2_VH. The Monaco DVH statistics converted into EQD_2_ were compared to the DVH statistics from EQD_2_VH by performing a Pearson correlation coefficient (PCC) to calculate the correlation among the datasets. This process was performed for each *R* and *S* value investigated. The correlation was performed on several structures in the head, neck, and thorax. The structures varied in volume (0.5–299 cm^3^) and dose received (1.1–73.75 Gy) to monitor EQD_2_VH's response to varying the interpolation settings for a variety of volumes and doses.

A gamma analysis was then performed to further investigate the differences between the DVHs, with the DVHs from Monaco being used as the reference DVH. The criteria for the gamma analysis were based on work by Ebert et al., which used a 95% pass rate with a volume‐difference criterion (Δ*V*) of 1% of the total volume, and dose‐to‐agreement criterion (Δ*D*) of 1% of the maximum DVH dose.^11^

### 2.3 Selecting patient studies

The evaluation of EQD_2_VH as a decision‐making tool was performed on case studies selected from patients undergoing EBRT during the May 2021 cyberattack. The criteria for the case studies were that their treatment time *T* was greater than the *T_delay_* of their tumor. Patients with a *T* less than their tumor's *T_delay_* would not experience rapid cell repopulation during their treatment and therefore were not selected for analysis. Patients undergoing a combination of EBRT and brachytherapy were also not selected to simplify the process of creating post treatment gap plans for the case studies.

The eligible patients were grouped by the categorization created by the RCR, which categorizes patients based on their prioritization for compensation. Category 1 (C1) patients, defined by the RCR as patients with rapidly growing tumors whose treatment gaps should not surpass 2 days, with a *T* > 1.5 × *T_delay_* were selected due to their rapid cell repopulation (*K* = 0.9 Gy day^−1^, *T_delay_* = 28 days) and their longer treatment times.^1^

Five C1 patients were chosen to evaluate the EQD_2_VH software as a decision‐making tool. The small cohort size was due to a limited patient population fitting the previous criteria. Of the five patients chosen, one patient was a lung cancer patient receiving 3D conformal radiotherapy and the remaining four patients were head‐and‐neck cancer patients receiving intensity‐modulated radiotherapy. The case studies were selected to evaluate how EQD_2_VH can be used for patients with conventional and nonconventional fractionation, with four of the five case studies receiving 2 Gy per fraction and one case study receiving 2.2 Gy per fraction. They also provide a range of variations in the timing of the treatment gap, with the pre‐gap treatment times ranging from 9 to 46 days. A summary of the case studies can be found in Table 2.

**TABLE 2:** Summary of the dose prescription details for five case studies chosen for the evaluation of EQD_2_VH

	Treatment site	*d* (Gy)	*N*	*T* (days)	PTV physical dose (Gy)	Treatment gap (days)
**Patient A**	**Left tonsil**					
Intended treatment		2.2	30	42	66	12
Pre‐gap dose		2.2	18	26	39.6	
Dose remaining		2.2	12	52	26.4	
**Patient B**	**Larynx**					
Intended treatment		2	35	50	70	12
Pre‐gap dose		2	31	46	62	
Dose remaining		2	4	59	8	
**Patient C**	**Vocal cord**					
Intended treatment		2	35	50	70	13
Pre‐gap dose		2	6	9	12	
Dose remaining		2	29	63	58	
**Patient D**	**Right lung**					
Intended treatment		2	30	44	60	12
Pre‐gap dose		2	12	18	24	
Dose remaining		2	18	51	36	
**Patient E**	**Parotid**					
Intended treatment		2	30	43	60	12
Pre‐gap dose		2	7	10	14	
Dose remaining		2	23	49	46	

Abbreviation: PTV, planning target volume.

### 2.4 Creating plans for dose compensation options

The analysis of compensation options for patients requiring dose compensation required the creation of revised treatment plans, comprising hypofractionation and acceleration fractions to account for repopulation effects in the PTV and to reduce *T* as recommended by the RCR.^1^ The term “revised plan” will be used to describe this approach in which the original treatment plan design is altered. The revised plans were made using the original treatment plans created for each patient at the beginning of their treatment. This was chosen instead of replanning or re‐optimizing the plans for consistency when evaluating EQD_2_VH. The revised plans for each patient, therefore, consisted of the same plan, with only the number of fractions or dose per fraction changing.

Post‐treatment gap schedules were created for each plan, following constraints recommended by the RCR^1^ and the National Cancer Control Programme (NCCP).^8^ The constraints were made in response to the COVID‐19 pandemic, accounting for prolonged treatment gaps and limitations in radiotherapy departments. The constraints recommended that twice‐daily fractionation not be performed on consecutive treatment days and recommended limiting the number of fractions to 6 per week.^1^, ^8^

**FIGURE 1:**
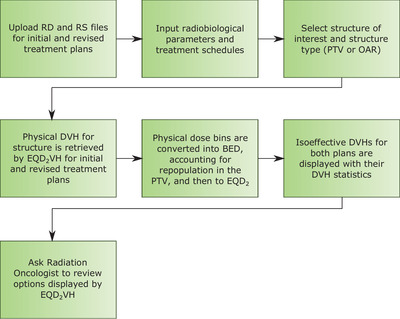
Workflow for using EQD_2_VH to compare revised plans with the patient's initial treatment pla

Each patient's revised treatment plan and schedule used a combination of twice‐daily fractionation, weekend treatments, and increasing *d* to reduce the effects of cell repopulation by shortening the overall treatment time and increasing the dose to the target volume. Revised plans were first made by using only accelerated fractions with a limit of 6 fractions per week to prevent an increase in normal tissue damage. Once the maximum number of weekend treatments and/or twice‐daily fractions using the prescription dose had been met, revised plans using hypofractionation were investigated. Figure 1 shows the workflow of the process used to evaluate the revised plans using EQD_2_VH.

## 3 RESULTS

### 3.1 Verification and output of software calculations

The analysis of varying the interpolation resolution *R* and the interpolation of segments *S* of the *dvhcalc.get_dvh* module revealed that setting both *R* and *S* to zero produced the best correlation between the Monaco and EQD_2_VH DVH statistics. The PCC performed on the DVH statistics in Table 3 resulted in an *r* of 0.99 for *R*, *S* = 0 showing a strong positive correlation among the datasets.

**TABLE 3:** Comparison of dose–volume histogram (DVH) statistics from EQD_2_VH and Monaco with *R*, *S* = 0. The *D_min_*, *D_mean_*, and *D_max_* are displayed in EQD_2_

ROI	Software	Volume (cm^3^)	*D_min_* (Gy)	*D_mean_* (Gy)	*D_max_* (Gy)
Brainstem	Monaco	20.91	1.15	8.94	37.42
	EQD_2_VH	21.06	1.25	9.23	38.17
Spinal cord	Monaco	49.81	0.56	10.10	41.23
	EQD_2_VH	49.57	0.12	9.93	41.15
Right optic nerve	Monaco	0.52	0.86	1.12	1.35
	EQD_2_VH	0.51	0.93	1.17	1.36
PTV 66 Gy	Monaco	299.83	53.57	64.63	71.7
	EQD_2_VH	299.35	57.83	67.16	73.75

Abbreviation: PTV, planning target volume.

**FIGURE 2:**
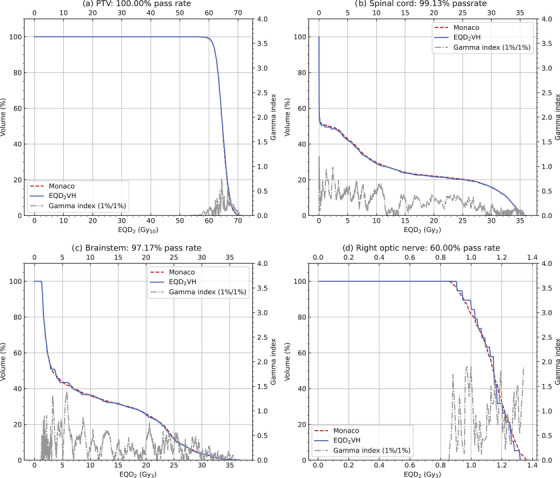
Dose–volume histogram (DVH) comparisons between EQD_2_VH and Monaco with *R*, *S* = 0 for the (a) 66 Gy planning target volume (PTV) (299.83 cm^3^), (b) spinal cord (49.81 cm^3^), (c) brainstem (20.91 cm^3^), and (d) right optic nerve (0.52 cm^3^). The gamma index is also displayed with a Δ*V* and Δ*D* = 1%.

Figure 2 shows the DVHs and gamma analysis for the PTV, brainstem, and right optic nerve for Patient A and spinal cord for Patient D. Although the PTV, brainstem, and the spinal cord met the criteria to pass the analysis, the right optic nerve failed with a passing rate of 60%. This failure is due to the staggered DVHs produced by EQD_2_VH with no interpolation present. The staggered DVHs for small volume structures (≤1 cm^3^) were a result of the volume of each structure being small relative to the dose grid. The dose at the center of each voxel in the TPS is used as the dose for the entire voxel in the EQD_2_VH DVH, and while this approximation works for larger structures, it results in a staggered appearance for smaller structures.

**FIGURE 3:**
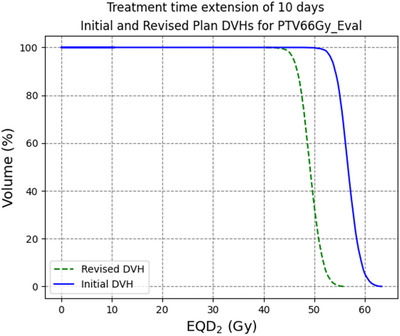
Graphical output of EQD_2_VH comparing the revised option for the 66 Gy planning target volume (PTV) against the initial treatment plan for Patient A

An example of the output of EQD_2_VH is shown in Figure 3, displaying the PTV DVH for the revised plan investigated for Patient A and their initial treatment plan, with a summary of this revised plan in Table 4. The units for BED and EQD_2_ in Table 4 display the α/αββ for each structure in the unit's subscript. The isoeffective DVH statistics expressed in EQD_2_ for the structure are printed for the initial and the revised plans, shown in Table 5. This allows for direct comparison between the two plans and demonstrates the improvement of the dose to the PTV for each revised plan, and the corresponding increase in dose to the OARs. Table 6 summarizes the uncompensated treatment plans used for Patients B–E in comparison to their intended treatment plans, accounting for repopulation effects. The presence of a negative post‐gap BED for Patient B in Table 6, who had completed 31 of 35 fractions before their treatment gap, is due to the dose lost to cell repopulation at the end of their treatment being greater than the dose they received in the four remaining fractions after their treatment gap.

**TABLE 4:** Patient A (66 Gy/33): plan comparison using EQD_2_VH in Figure 3

DVH statistics	Physical dose to PTV (Gy)	*d* (Gy)	*N*	*T* (days)	BED corrected for repopulation (Gy_10_)	EQD_2_ corrected for repopulation (Gy_10_)	Dose lost to repopulation (Gy_10_)
Initial plan	66	2.2	30	42	66.6	55.5	12.6
Pre‐gap	39.6	2.2	18	26	47.5	39.6	0.0
Post‐gap	26.4	2.2	12	52	10.1	9.6	21.6
Uncompensated plan	66.0	2.2	30	52	57.6	48	21.6
Difference (intended‐uncompensated)					9 (Gy_10_)	7.7 (Gy_10_)	

Abbreviations: BED, biologically effective dose; DVH, dose–volume histogram; PTV, planning target volume.

**TABLE 5:** Dose–volume histogram (DVH) statistics for planning target volume (PTV) expressed as EQD_2_ incorporating repopulation correction printed by EQD_2_VH when comparing plans in Figure 3 for Patient A

DVH statistics for PTV expressed as EQD_2_	Initial plan (Gy_10_) (including repopulation correction)	Revised plan (Gy_10_) (including repopulation correction)
Max dose	63.3	56.5
Min dose	47.3	40.6
Mean dose	56.7	49.9
*D*_100%_	47.3	40.6
*D*_98%_	52.6	45.0
*D*_95%_	53.5	46.7
*D*_2%_	60.9	54.2
*D*_50%_	56.6	49.9
*D*_2cc_	61.9	55.1

Abbreviations: DVH, dose–volume histogram; PTV, planning target volume.

**TABLE 6:** Patient B–E treatment plan comparison between initial and uncompensated treatment plans

DVH statistics	Physical dose to PTV (Gy)	*d* (Gy)	*N*	*T* (days)	BED corrected for repopulation (Gy_10_)	EQD_2_ corrected for repopulation (Gy_10_)	Dose lost to repopulation (Gy_10_)
**Patient B**							
Initial plan	70	2	35	50	64.2	53.5	19.8
Pre‐gap	62	2	31	46	58.2	48.5	16.2
Post‐gap	8	2	4	59	−2.1	−1.8	27.9
Uncompensated plan	70	2	35	59	56.1	46.6	27.9
Difference (intended‐uncompensated)					8.1 (Gy_10_)	6.7 (Gy_10_)	
**Patient C**							
Initial plan	70	2	35	50	64.2	53.5	19.8
Pre‐gap	12	2	6	9	14.4	12	–
Post‐gap	58	2	29	63	38.1	31.8	31.5
Uncompensated plan	58	2	29	63	52.5	43.8	31.5
Difference (intended‐uncompensated)					11.7 (Gy_10_)	9.7 (Gy_10_)	
**Patient D**							
Initial plan	60	2	30	44	57.6	48	14.4
Pre‐gap	24	2	12	18	28.8	24	–
Post‐gap	36	2	18	51	23.4	19.5	19.8
Uncompensated plan	60	2	30	51	52.2	43.5	19.8
Difference (intended‐uncompensated)					5.4 (Gy_10_)	4.5 (Gy_10_)	
**Patient E**							
Initial plan	60	2	30	43	58.5	48.8	13.5
Pre‐gap	14	2	7	10	16.8	14	–
Post‐gap	46	2	23	49	36.3	30.3	18.9
Uncompensated plan	60	2	30	49	53.1	44.3	18.9
Difference (intended‐uncompensated)					5.4 (Gy_10_)	4.5 (Gy_10_)	

Abbreviations: BED, biologically effective dose; DVH, dose–volume histogram; PTV, planning target volume.

### 3.2 Comparison with current method

**FIGURE 4:**
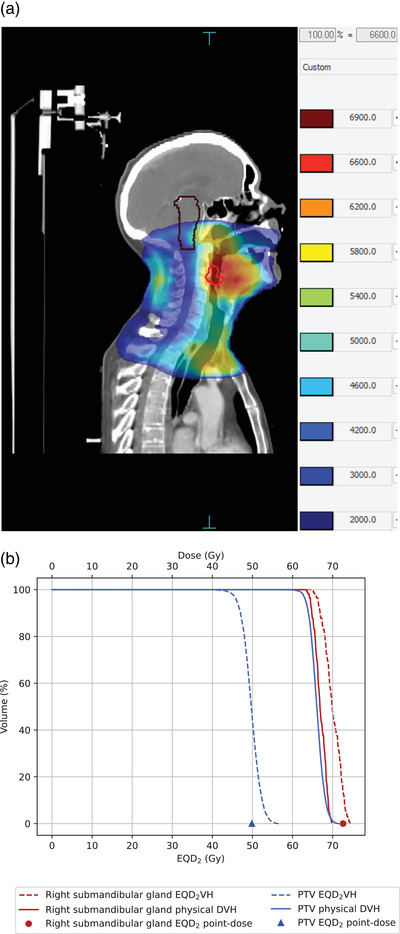
(a) Sagittal view of Patient A's treatment plan, with the planning target volume (PTV), outlined in red (b) comparison of the point‐dose calculation method, physical dose–volume histogram (DVH), and EQD_2_VH for the PTV and the right submandibular gland

Current treatment gap calculations represent each compensation option with a point‐dose. Figure 4 compares the physical DVH, radiobiological DVH, and the point‐dose approach for the PTV and the right submandibular gland for Patient A. The point‐dose values were calculated using the calculation methods recommended by the RCR. This demonstrates how condensing each treatment option into a single point oversimplifies the effects of the treatment plan to each structure.

In contrast, the radiobiological DVHs from EQD_2_VH provide an individual, 2D representation of each treatment option to each structure. It accounts for dose‐gradients in each structure by using the variable dose per fraction (Equations 6 and 7) and provides a quantitative analysis of each plan for comparison with dose constraints.

### 3.3 Clinical applications of EQD_2_VH

The revised plans created for Patients A–E are summarized in Table 7. The revised plans were created to reduce the *T* by using twice‐daily fractionation and weekend treatments following the six fractions per week constraint recommended by the NCCP,^8^ and using hypofractionation to reduce *T* and compensate for dose lost to cell repopulation.

**TABLE 7:** Revised plans created for Patient A–E and evaluated with EQD_2_VH, showing the number of fractions *N*, dose per fraction *d*, total physical dose *D*, treatment time *T*, number of days with twice‐daily fractions *TD*, the dose lost to cell repopulation for each *T*, the *D*_98%_ for each plan accounting for repopulation, and the difference in the *D*_98%_ between the intended and revised plans

Revised plan	*N*	*d* (Gy)	Physical dose *D* (Gy)	*T* (days)	*TD* (days)	Dose lost to repopulation (Gy_10_)	Cumulative EQD_2_ *D*_98%_ corrected for repopulation (Gy_10_)	Diff. from *D*_98init_ (Gy_10_)
**Patient A**	**PTV = left tonsil**							
Intended treatment	30	2.2	66.0	42	0	12.6	51.8	–
No compensation	12	2.2	26.4	52	0	21.6	44.3	−7.5
Revised plan 1	12	2.2	26.4	51	1	20.7	45.0	−6.8
Revised plan 2	12	2.2	26.4	47	2	17.1	48.1	−3.7
Revised plan 3	12	2.5	30.0	51	1	20.1	49.1	−2.7
Revised plan 4	12	2.5	30.0	50	2	19.8	50.7	−1.1
**Patient B**	**PTV = larynx**							
Intended treatment	35	2.0	70.0	50	0	19.8	48.8	–
No compensation	4	2.0	8.0	62	0	30.6	39.8	−9.0
Revised plan 1	4	2.0	8.0	59	1	27.9	42.0	−6.8
Revised plan 2	4	2.2	8.8	52	0	21.6	40.6	−8.2
Revised plan 3	**5**	2.5	12.5	59	2	27.9	46.7	−2.1
**Patient C**	**PTV = vocal cord**							
Intended treatment	35	2.0	70.0	50	0	19.8	50.6	–
No compensation	29	2.0	58.0	63	0	31.5	37.9	−12.7
Revised plan 1	29	2.0	58.0	62	0	30.6	38.7	−11.9
Revised plan 2	29	2.0	58.0	56	5	25.2	43.2	−7.4
Revised plan 3	29	2.1	60.9	56	0	25.2	48.6	−2.0
Revised plan 4	29	2.2	60.9	54	4	23.4	50.1	−0.5
**Patient D**	**PTV = right lung**							
Intended treatment	30	2.0	60.0	44	0	14.4	32.8	–
No compensation	18	2.0	36.0	55	0	24.3	23.8	−9.0
Revised plan 1	18	2.0	36.0	50	1	19.8	27.6	−5.2
Revised plan 2	18	2.2	39.6	55	0	24.3	26.8	−6.0
Revised plan 3	18	2.0	36.0	48	4	18.0	29.0	−3.8
Revised plan 4	18	2.2	39.6	49	4	18.9	31.3	−1.5
**Patient E**	**PTV = parotid**							
Intended treatment	30	2.0	66.0	43	0	13.5	44.7	–
No compensation	23	2.0	46.0	54	0	23.4	35.7	−9.0
Revised plan 1	23	2.0	46.0	49	1	18.9	39.5	−5.2
Revised plan 2	23	2.1	48.3	49	1	18.9	41.9	−2.8
Revised plan 3	23	2.0	46.0	43	4	13.5	43.7	−1.0
Revised plan 4	23	2.2	50.6	50	0	19.8	43.6	−0.7

Abbreviation: PTV, planning target volume.

**FIGURE 5:**
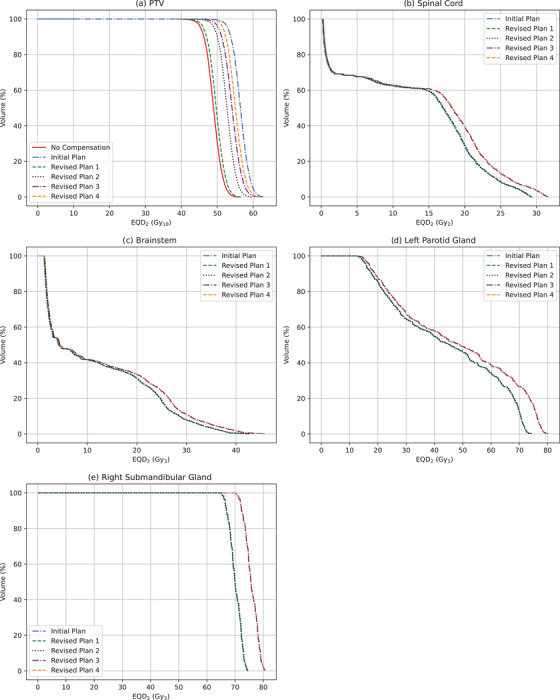
Patient A's (a) planning target volume (PTV), (b) spinal cord, (c) brainstem, (d) left parotid gland, and (e) right submandibular gland comparing the initial treatment plan for the patient against the revised plans

The DVH statistics, expressed in EQD_2_ with repopulation applied, for each plan provided in Table 7 were obtained using EQD_2_VH. A direct comparison was provided by EQD_2_VH between each plan being investigated for the patient against their intended uninterrupted treatment plan for each structure. Figure 5 shows the DVHs in EQD_2_ for Patient A and demonstrates the effects of each plan on the patient's PTV and OARs. The initial plan, revised plan 1, and revised plan 2 consisted of the same fractionation (2.2 Gy in 12 fractions) with their use of twice‐daily fractionation being the only change. This is also the case for revised plan 3 and 4 (2.5 Gy in 12 fractions). This results in the OAR DVHs being approximately the same, with only minor differences due to twice‐daily fractionation. This shows EQD_2_VH's capabilities as an OAR monitoring tool when evaluating treatment plans for patients after a treatment gap, as it shows a direct comparison of each plan's DVH against what was initially intended for the patient.

**FIGURE 6:**
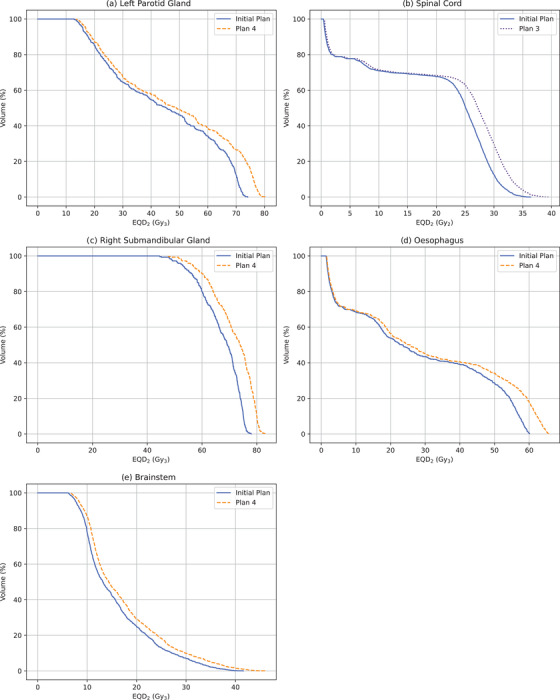
Organs at risk (OAR) dose–volume histogram (DVH) comparisons for (a) Patient A left parotid gland, (b) Patient B's spinal cord, and (c) Patient C's right submandibular gland, (d) Patient D's oesophagus, and (e) Patient E's brainstem showing their initial treatment plan against the revised plan with the highest dose to the planning target volume (PTV)

Figure 6 shows OAR DVHs for each patient comparing their initial treatment plan against the revised plan that results in the largest increase in dose to the PTV, as seen in Table 7. Despite these revised plans not fully regaining the dose to the PTV, they result in substantial increases in dose to the OARs. The *D_max_* to the spinal cord for Patient C increased from 44.8 to 48.2 Gy, only 1.8 Gy from its dose constraint of 50 Gy. Due to their close proximity to the PTV, Patient A's left parotid gland and Patient C's right submandibular gland received *D_mean_* of 48.06 and 70.5 Gy in EQD_2_, respectively, when using these revised plans.

### 3.4 Twice‐daily fractionation

The effects of sublethal damage to unrepaired normal tissue are accounted for in EQD_2_VH calculations; therefore, it can be used to evaluate plans using twice‐daily fractionation a minimum of 6 h apart as a compensation method. The increase in the *D*_2%_ to the brainstem, left submandibular gland, and the spinal cord in Table 8 shows the increase in dose to the normal tissue when using twice‐daily fractionation.

**TABLE 8:** Dose–volume histogram (DVH) statistics in EQD_2_ showing the increase in dose when increasing treatment days with twice‐daily fractionation *TD* for Patient A

Structure	*TD* = 0	*TD* = 1	*TD* = 2	*TD* = 3	*TD* = 4
Brainstem *D*_2%_ (Gy_3_)	35.4	35.6	35.8	36.0	36.2
Left submandibular gland *D*_2%_ (Gy_3_)	63.6	63.8	64.1	64.4	64.6
Spinal cord *D*_2%_ (Gy_2_)	28.6	28.7	28.8	28.9	29.1

The repair half time *T*_1/2_ was assumed to be 2 h. This was the median repair halftime for normal tissue that ranges from 1 to 3 h.^12^ It was also assumed that the interfraction interval *m* was 6 h, which is the minimum time recommended by the RCR.^2^ Using these values for *T*_1/2_ and *m*, the *h* for the OARs was 0.125^5^. For the tumor, the effects of sublethal damage to unrepaired tissue were negligible after a 6 h period; therefore, *h* was taken to be 0.

The apparent increase in the radiobiological dose is minimal relative to the cumulative dose, particularly in low‐dose regions. The spinal cord's *D*_2%_ increased by only 0.5 Gy after 4 days of twice‐daily treatments. The increase in the *D*_2%_ for the brainstem was 0.74 and 1.04 Gy for the left submandibular gland. Despite the low increase in cumulative dose to the OARs, twice‐daily fractionation can be an intensive compensation option and should not be used excessively. The DAHANCA trials evaluated the effectiveness of 6 versus 5 fractions per week for radiotherapy patients and found that while the overall survival remained the same, there was an increase in acute toxicities for patients undergoing 6 fractions per week.^13^

## 4 DISCUSSION

### 4.1 Software calculation

The use of interpolation in EQD_2_VH was investigated using structures with varying dose levels and volume sizes. The effects of interpolation were greater for small volume structures (<1 cm^3^). The staggered appearance of the DVH plots for small volume structures was improved greatly when increasing *R* and *S* resulting in smoother curves in the DVH; however, this also resulted in apparent changes in the volume of the structure provided in the DVH statistics. It also resulted in increases in dose for medium‐to‐large volume structures (>20 cm^3^) and proved to be incompatible with certain structures, with Dicompyler being unable to retrieve the DVH information from the spinal cord of the head‐and‐neck plans. To prevent future errors from occurring and the volume of the structure changing, it was decided to proceed with no interpolation of the data. With *R*, *S* = 0, the PCC signified a strong correlation between the EQD_2_VH and Monaco DVH statistics.

The correlation between EQD_2_VH and Monaco was further investigated with a gamma analysis for *R*, *S* = 0. The absence of interpolation resulted in a lower pass rate for small structures, as seen with the right optic nerve in Figure 2, which only had a 60% pass rate. Although the criteria for the gamma analysis (Δ*V* = 1% of the total volume and Δ*D* = 1% of the maximum DVH dose) were low in comparison to the Δ*D* = 3% typically used in dose distribution measurements,^14^ this increased the sensitivity of the analysis to minor differences between the DVHs.

### 4.2 Evaluation of EQD_2_VH

The 2D representation of each individual structure in EQD_2_VH provides a more in‐depth analysis than the 1D point‐dose calculation method. Using the 1D calculation method, each compensation option is limited to a single‐dose point for the PTV and OARs in the treatment plan. The physical dose distribution and the dose gradients present in each OAR are not accounted for and a significant overestimation of the dose received by the whole OAR is obtained. The visual and quantitative components of EQD_2_VH can aid radiation oncologists in deciding which treatment option is most suitable for a patient after a treatment gap.

The use of EQD_2_VH as a decision‐making tool was investigated using case studies. The effects of varying compensation options on the PTV for Patients A–E were investigated using EQD_2_VH, along with the impact that the most successful compensation option had on the OARs. The rate of cell repopulation in normal tissue is slower than tumor tissue, and the dose lost to cell repopulation is negligible within the timeframe of a radiotherapy treatment. Therefore, compensating for dose lost to the PTV will result in an increase in dose to the OARs. EQD_2_VH provides a means of monitoring each OAR in the patient's plan to minimize further increases to their dose. The ability to compare each revised plan to the patient's initial treatment plan in EQD_2_VH provides a way of monitoring the dose to the PTV and OARs to reduce acute toxicities and determine the most appropriate treatment option. EQD_2_VH also provides a method of evaluating treatment‐gap lengths and determining when to move patients to a different hospital should radiotherapy services not have resumed. The patient's starting and proposed finishing treatment dates can be input into EQD_2_VH that allows the user to monitor the effects of different gap lengths on the patient's treatment plan.

Although there is commercially available 3D dose‐distribution software that can provide a voxel‐based radiobiological dose distribution, there are benefits to using the 2D‐based EQD_2_VH as a clinical aid when evaluating compensated plans. EQD_2_VH can account for changes in the overall treatment time and sublethal damage in normal tissue, which may not be accounted for in all 3D EQD_2_ dose distributions. EQD_2_VH also does not need to be connected to a hospital's network to function and can be stored using cloud storage that allows it to be used should a hospital's servers be inaccessible during a cyberattack.

### 4.3 Limitations of EQD_2_VH

Limitations of EQD_2_VH lie in the uncertainties in the radiobiological parameters used in the calculations. The rate of cell repopulation can vary from patient‐to‐patient, the stage of the cell cycle, and the type of tumor. Assuming *K* to be constant at 0.9 Gy day^−1^ for each C1 patient does not account for these variations in cell repopulation. The use of 0.9 Gy day^−1^ is recommended by the RCR for treatment gap calculations; however, an analysis from the RTOG 9003 trials^15^ shows that it can vary between 0.94 and 0.99 Gy day^−1^.^16^, ^17^ Similar variations were found for *T_delay_*, which can vary between patients and tumor types in the head, neck, and lung. Clinical trials have measured *T_delay_* to be 26 and 29 days.^18^ With this information, the RCR recommended a *T_delay_* of 28 days. Additional guidance from the RCR in April and May 2020, in response to the COVID‐19 pandemic, produced a table of suggested parameters, including *K* and *T_delay_* for various tumor types across anatomical sites.^19^, ^20^

The repair halftime for all OARs was assumed to be 2 h, which was the median *T*_1/2_ found by Pop et al.^12^ This assumption was made for simplification; however, it results in an underestimation of repair in structures, such as the kidney (*T*_1/2_ = 1.29 ± 0.16 h^21^), and an overestimation of repair for the heart (*T*_1/2_ ≥ 3 h^22^). The use of a single *T*_1/2_ for an organ can in itself be a simplification due to the repair kinetics of organs. Previous studies have found two repair half times for the spinal cord comprising short and long components.^23^ It was also assumed that the interfraction interval was 6 h for all calculations; however, in practice this time interval would vary given machine availability and delays from other patients. An extension onto the interfraction interval would decrease *h* due to an increase in normal tissue repair.^2^

The use of DVHs also limits the spatial information provided by EQD_2_VH. DVHs show a 2D representation of a 3D dose distribution; therefore, the location of the dose within each structure is lost in the conversion process.^24^ The presence and location of hot or cold spots in the structures are unknown when looking at the DVHs. They should not be used as the sole means of plan evaluation and should be used in conjunction with other plan evaluation tools.

## 5 CONCLUSION

Prolonged treatment gaps that occurred on a large scale during the COVID‐19 pandemic and the May 2021 HSE cyberattack highlighted the need for improved calculation methods when designing treatment compensation plans. The EQD_2_VH method was created to provide a 2D representation of the effects of each compensation option to each individual structure in a patient's treatment plan. It converts the physical dose calculated by the TPS in the DVH dose bins for each structure into EQD_2_ while accounting for the effects of cell repopulation in tumor tissue and damage to unrepaired normal tissue during closely spaced fractions.

The results demonstrate the role EQD_2_VH can play in treatment gap calculations for dose compensation and it is used as a decision‐making tool when deciding the most appropriate compensation option for a patient. It provides radiobiological DVHs that account for nonuniform dose distributions, and a direct visual and quantitative comparison between the plans being investigated and the intended plan prescribed to the patient initially. Key DVH statistics are provided for each plan that aids in monitoring dose and comparing to dose constraints.

## CONFLICT OF INTEREST

The authors declare that there is no conflict of interest that could be perceived as prejudicing the impartiality of the research reported.

## AUTHOR CONTRIBUTIONS

Conception and design: Katie O'Shea, Margaret Moore, and Linda Coleman.

Acquisition, collection, and assembly of data: Katie O'Shea, Louise Fahy, Margaret Moore, and Linda Coleman.

Data analysis and interpretation: Katie O'Shea and Margaret Moore.

Drafting of the work, revising it critically for important, intellectual, content, and paper writing: Katie O'Shea, Louise Fahy, Margaret Moore, Mark J. Foley, and Christoph Kleefeld.

Final approval of the paper: Katie O'Shea, Louise Fahy, Margaret Moore, Linda Coleman, Mark J. Foley, and Christoph Kleefeld.

The integrity of the work as a whole, from inception to published article: Katie O'Shea and Margaret Moore.

Agreement to be accountable for all aspects of the work in ensuring that questions related to the accuracy or integrity of any part of the work are appropriately investigated and resolved: Katie O'Shea, Margaret Moore, and Linda Coleman.
